# Parallel processing model for low-dose computed tomography image denoising

**DOI:** 10.1186/s42492-024-00165-8

**Published:** 2024-06-12

**Authors:** Libing Yao, Jiping Wang, Zhongyi Wu, Qiang Du, Xiaodong Yang, Ming Li, Jian Zheng

**Affiliations:** 1 School of Biomedical Engineering (Suzhou), Division of Life Sciences and Medicine, University of Science and Technology of China, Hefei, 230026 China; 2 Medical Imaging Department, Suzhou Institute of Biomedical Engineering and Technology, Chinese Academy of Sciences, Suzhou, 215163 China

**Keywords:** Deep learning, Low-dose computed tomography, Multi-encoder deep feature transformation, Multisource denoising

## Abstract

Low-dose computed tomography (LDCT) has gained increasing attention owing to its crucial role in reducing radiation exposure in patients. However, LDCT-reconstructed images often suffer from significant noise and artifacts, negatively impacting the radiologists’ ability to accurately diagnose. To address this issue, many studies have focused on denoising LDCT images using deep learning (DL) methods. However, these DL-based denoising methods have been hindered by the highly variable feature distribution of LDCT data from different imaging sources, which adversely affects the performance of current denoising models. In this study, we propose a parallel processing model, the multi-encoder deep feature transformation network (MDFTN), which is designed to enhance the performance of LDCT imaging for multisource data. Unlike traditional network structures, which rely on continual learning to process multitask data, the approach can simultaneously handle LDCT images within a unified framework from various imaging sources. The proposed MDFTN consists of multiple encoders and decoders along with a deep feature transformation module (DFTM). During forward propagation in network training, each encoder extracts diverse features from its respective data source in parallel and the DFTM compresses these features into a shared feature space. Subsequently, each decoder performs an inverse operation for multisource loss estimation. Through collaborative training, the proposed MDFTN leverages the complementary advantages of multisource data distribution to enhance its adaptability and generalization. Numerous experiments were conducted on two public datasets and one local dataset, which demonstrated that the proposed network model can simultaneously process multisource data while effectively suppressing noise and preserving fine structures. The source code is available at https://github.com/123456789ey/MDFTN.

## Introduction

Computed tomography (CT) is a widely used medical imaging technique in clinical practice. Owing to its fast scanning speed, high image resolution, and ability to display fine lesions or tissue structures, it plays an increasingly important role in diagnosing lung diseases, neurological lesions, and cardiovascular abnormalities [1]. However, numerous clinical studies have demonstrated that superimposed X-ray scans can cause radiation damage to patients’ normal tissues and increase the risk of cancer [2–5]. The reduction in radiation dose during X-ray scanning has garnered significant attention from scholars. Various approaches, such as reducing the tube current and tube voltage and increasing the helical pitch, have been explored to lower the radiation dose of X-ray CT. However, these methods often produce images with significant noise and artifacts. Therefore, the development of low-dose CT (LDCT) imaging methods to enhance image quality is imperative [6].

In general, a practical method for enhancing the overall image quality is to establish a reasonable model that accurately simulates noisy anatomy. Prior to the widespread adoption of deep learning (DL) in low-dose CT imaging, researchers primarily concentrated on three approaches to noise reduction: sinogram-domain filtering methods [7–10], iterative reconstruction methods [11–16], and post-processing methods [17–20]. First, to restore the local structure of the sinogram domain, a distinct local filter kernel is generated for each input measurement. This approach aims to preserve edge information while reducing noise in the reconstructed image. Examples of such filters include the noise-adaptive bilateral [9] and structure-adaptive sinogram filters [10]. Iterative reconstruction methods are widely used [11, 12]. These methods combine prior information from the image domain with the data characteristics of the projection domain to reconstruct high-quality CT images. For example, total variation regularization [13] utilizes the L1 norm of the image gradient as an image constraint, which effectively suppresses noise in LDCT image reconstruction. To enhance the preservation of edge information, Yu et al. [14] introduced a method that utilizes the L0 norm as a regularization constraint and employs variable separation and alternate directions to solve nonconvex optimization problems. Other approaches such as dictionary learning methods [16] aim to extract local structural information from image patches and achieve high-quality image reconstruction. In comparison to the previous two methods, post-processing methods [19, 20] can be directly applied to reconstructed CT images without being influenced by the equipment or scanning system. Although these methods have been extensively used in various clinical imaging scenarios, there remain limitations in terms of detailed reconstructions.

With the increasing popularity of artificial intelligence technology, reconstruction methods using DL have been widely employed in LDCT and have demonstrated remarkable imaging performance [21–32]. Chen et al. [25] introduced a self-encode-decode network (RED-CNN) that demonstrates the potential of DL to reduce image noise related to anatomical structures. Shan et al. [27] proposed a modularized adaptive processing neural network (MAP-NN) for process-oriented image denoising. Du et al. [28] developed a modularized iterative network framework to address the issues of detail loss and gradient disappearance in MAP-NNs. Other researchers have explored self-supervised and unsupervised methods for LDCT image noise suppression [33–36]. To address the domain-shift image-denoising problem, Wang et al. [37] utilized noise estimation and transfer learning to propose a domain-adaptive denoising network, which showed promising results in addressing the issue of varying data distributions in clinical LDCT. Yang et al. [38] introduced a hypernetwork-based, physics-driven, personalized federated learning (FL) approach (HyperFed) to address domain shifts and privacy issues. Li et al. [39] adopted a Gaussian mixture model (GMM) to quantify the noise distribution in CT images. Based on this quantification, they proposed an unsupervised GMM-UNNET method to mitigate the issues related to noise distribution drift arising from varying scanning protocols. Li et al. [40] introduced a generative adversarial network with noise-encoding transfer learning, which effectively generates paired clinical LDCT images to address the domain-adaptation issue. Moreover, several researchers have integrated DL with iterative reconstruction techniques to further enhance the quality of LDCT images [41–45]. Zhang et al. [44] introduced a comprehensive learning-enabled adversarial reconstruction method for enhancing the structural fidelity and visual perception of LDCT images. Hu et al. [45] combined iterative optimization with DL using residual learning to improve the convergence and versatility of the LDCT.

**Fig. 1:**
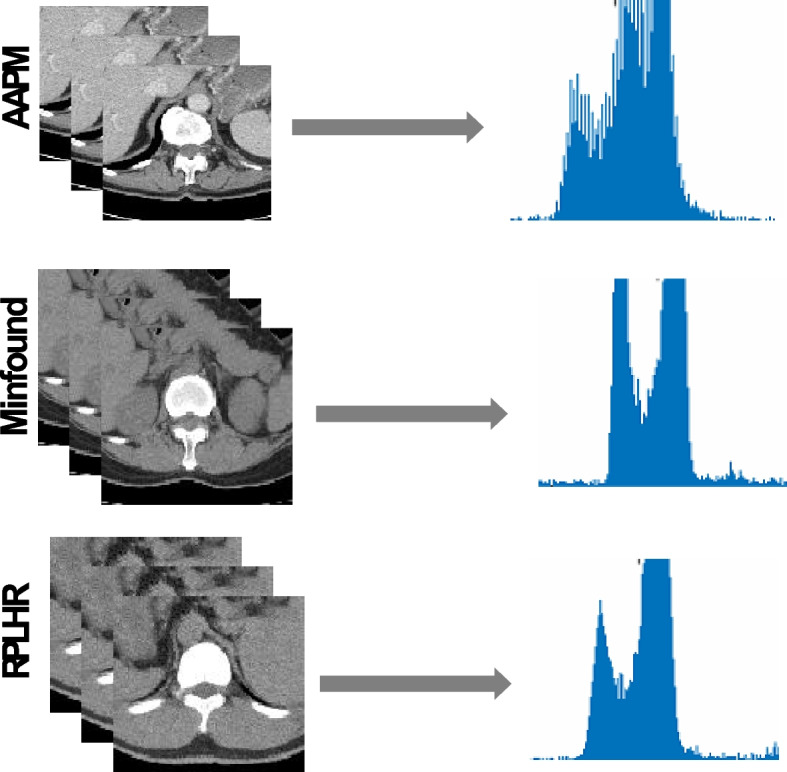
CT image data distribution of different manufacturers

Although these methods can enhance the quality of LDCT images with uncertain noise, they have a limited ability to simultaneously process LDCT images from multiple sources and rely solely on a specific dataset. CT devices from various manufacturers employ different low-dose scanning protocols, hardware devices, and data processing procedures, resulting in diverse distributions of LDCT images, as illustrated in Fig. 1. Consequently, researchers typically conduct multisource low-dose CT denoising with continual learning or domain-adaptive learning [46, 47]; however, this results in lower efficiency or catastrophic forgetting problems. Despite the extensive use of DL models in medical imaging, the inherent scarcity and imbalance of medical datasets significantly hinder their performance of DL models. Therefore, it is crucial to design a learning-once model that can effectively handle multisource LDCT images and successfully address the challenges associated with small datasets. The objective of this study is to develop a robust model capable of simultaneously handling multisource datasets and surpassing the performance of individual models through continual learning.

In this study, a parallel-processing model called multi-encoder deep feature transformation network (MDFTN) is introduced, which is designed to denoise multisource, low-dose CT images. First, a multibranch parallel encoder is utilized to extract diverse features from multisource datasets. A deep feature transformation module (DFTM) then compresses these features into a shared feature space, enabling the mutual enhancement of features from different datasets. Finally, each decoder performs an inverse operation for multisource loss estimation and generates multisource LDCT images. During backward propagation in network training, joint loss functions are employed to calculate the gradient of each layer and update all the network weights accordingly. Through collaborative training, the proposed MDFTN leverages the complementary advantages of multisource data distribution to enhance its adaptability and generalization.

The remainder of this article is organized as follows: Methods section presents a thorough description of the method employed, including the network framework and DFTM. In Results section, the effectiveness of the proposed method is assessed using both multisource simulation and real-world clinical datasets. The experimental results are detailed and relevant ablation experiments are discussed in this section. Finally, a comprehensive discussion and conclusions are presented in Discussion.

## Methods

### CT denoising model

Regarding deep-learning-based LDCT denoising methods, the denoising model is considered a mapping function that transforms the LDCT input into a normal-dose CT (NDCT) output. Let $x \in LDCT^{{H{ \times }W}}$ and $y \in NDCT^{{H{ \times }W}}$ represent the LDCT and NDCT images, respectively, and $W$ and $H$ denote the width and height of the image matrix, respectively. Thus, the relationship between the two can be expressed as follows:

1 $$x = y + m$$

$m$ is a complex degradation process that primarily involves quantum and electronic noises, among other factors. The CT denoising model is considered an inverse problem, in which a deep network model is used to construct a feature map $f$, given an LDCT image, to estimate the NDCT image. This relationship is represented by Eq. (2).

2 $$f:x \to y$$

In theory, DL-based methods have the potential to enhance denoising performance by extracting more comprehensive feature distributions from the network model. This allows the estimation of network parameters using DL techniques.

3 $$\mathop {\min }\limits_{f} \left\| {f(x) - y} \right\|_{2}^{2}$$

### Network framework overview

**Fig. 2:**
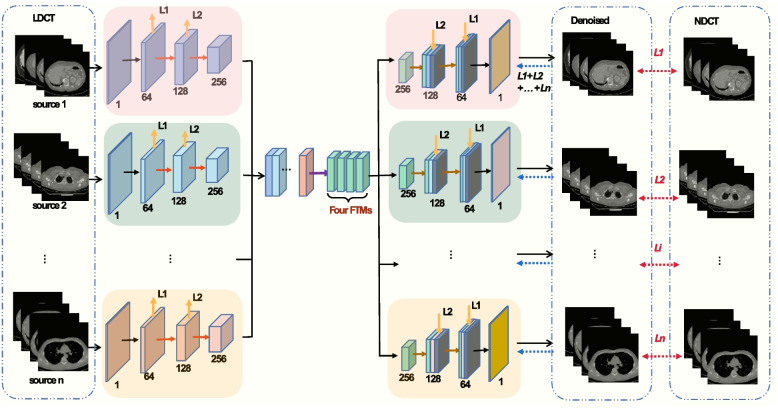
Overall framework of the proposed MDFTN network. The overall network consists of multiple encoders and decoders, along with a DFTM

Inspired by the excellent performance of refs. [48–50], a novel MDFTN is proposed with collaborative training for denoising multisource low-dose CT images, as illustrated in Fig. 2. The proposed MDFTN consists of multiple encoders and decoders along with a DFTM, each of which is responsible for a specific task. First, during forward propagation in network training, low-dose images from multisource datasets are fed into CNN-based encoders. These encoders independently extract diverse levels of image features in parallel rom their respective data sources. Encoders play a pivotal role in simultaneously extracting data from diverse sources, thereby mitigating the risk of forgetting the data. This capability is particularly beneficial when dealing with multisource datasets, as it addresses the challenges associated with continuous learning or domain-adaptive learning and enhances the efficiency and effectiveness of data processing. Second, the DFTM combines and compresses the various features into a shared feature space. This shared feature space allows the extraction of consistent features from different data sources, thereby complementing the features of different datasets. Finally, each decoder performs an inverse operation for multisource loss estimation to generate distinct high-resolution CT images from the shared features. During backward propagation in network training, joint loss functions are employed to calculate the gradient of each layer and update all the network weights accordingly. Through collaborative training, the proposed MDFTN leverages the complementary advantages of multisource data distribution to enhance its adaptability and generalization.

#### Encoders and decoders

**Fig. 3:**
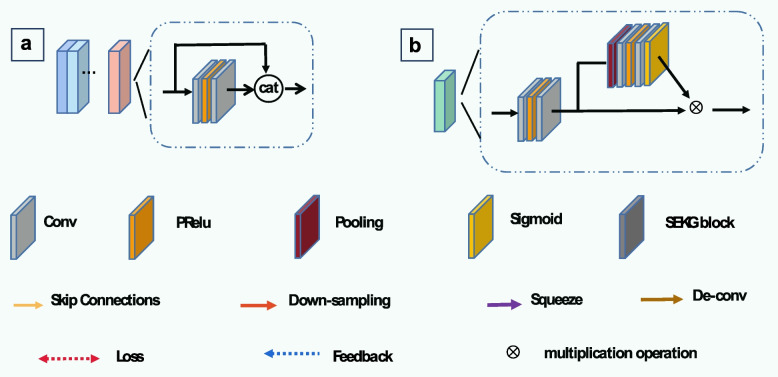
Detailed description of the proposed MDFTN network. **a** Residual block; **b** FTM. MDFTN consists of four FTMs

As shown in Fig. 2, multisource image reconstruction tasks are performed with multiple parallel encoders. Each image is processed by its corresponding DL mapping function. The encoder and decoder are modified based on the U-net network [51], performing two downsampling and two upsampling operations. Furthermore, encoders have similar network structure but receive different inputs, which are LDCT images from multisource datasets. Here, residual blocks (Fig. 3a) are used instead of cascading convolutional layers in the classical U-net network [51]. 1 ${ \times }$ 1 convolution is adopted to adaptively fuse a series of features at different levels, and 3 ${ \times }$ 3 convolution is exploited to extract global information. To expedite network training, the encoders are designed to simultaneously learn and reconstruct information. It is assumed that the degraded LDCT images of different sources $$\left( {\begin{array}{*{20}c} {x_{a}^{1} ,x_{a}^{2} ,...,x_{a}^{m} } \\ \begin{gathered} x_{{\text{b}}}^{1} ,x_{b}^{2} ,...,x_{b}^{m} \\ \cdot \cdot \cdot \\ \end{gathered} \\ {x_{n}^{1} ,x_{n}^{2} ,...,x_{n}^{m} } \\ \end{array} } \right) \in R^{{H{ \times }W}}$$ and features extracted by encoders are $$\left( \begin{gathered} \begin{array}{*{20}c} {F_{a} } \\ {F_{b} } \\ { \cdot \cdot \cdot } \\ \end{array} \hfill \\ F_{n} \hfill \\ \end{gathered} \right) \in R^{{\frac{H}{4}{ \times }\frac{W}{4}{ \times }256}}$$. The multi-parallel coding task is defined as Eq. (4), where $\theta_{1}$,$\theta_{2}$,…,$\theta_{n}$ are the parameters of encoders $E_{1}$,$E_{2}$,…,$E_{n}$.

4 $$\left( {\begin{array}{*{20}c} {F_{a} } \\ \begin{gathered} F_{b} \\ \cdot \cdot \cdot \\ \end{gathered} \\ {F_{n} } \\ \end{array} } \right) = \left( {\begin{array}{*{20}c} {E_{1} (x_{a}^{1} ,x_{a}^{2} ,...,x_{a}^{m} ,\theta_{1} )} \\ \begin{gathered} E_{2} (x_{{\text{b}}}^{1} ,x_{b}^{2} ,...,x_{b}^{m} ,\theta_{2} ) \\ \cdot \cdot \cdot \\ \end{gathered} \\ {E_{n} (x_{n}^{1} ,x_{n}^{2} ,...,x_{n}^{m} ,\theta_{n} )} \\ \end{array} } \right)$$

During network training, downsampling is achieved through an average pooling operation, whereas upsampling is achieved through a transposed convolution operation. During the decoding process, deep features from different image encoders are mapped and connected to the middle layer of the corresponding decoder, as shown in Fig. 2. Here, the decoders incorporate spatially enhanced kernel generation (SEKG) modules [52], which perform spatial attention using a simple 3 ${ \times }$ 3 depthwise separable convolution. In addition, they include channel attention, which utilizes average pooling and convolution mapping layers to generate the information weight $\Theta$. The dimensions of the $\Theta$ are (*n* ${ \times }$ *c* ${ \times }$ *k* ${ \times }$ *k*)${ \times }$ *h* ${ \times }$ *w*, where *n*, *c*, *k*, *h*, and *w* represent the batch size, number of channels, convolution kernel, height, and width, respectively. The unfold operation extracts a sliding local area $F_{unfold} { \in }{\mathbf{\mathbb{R}}}^{{c \times k^{2} \times h \times w}}$ from the input feature *x* with a patch size of *k* = 3 and a stride of *s* = 1. The new regional feature $F_{unfold} { \in }{\mathbf{\mathbb{R}}}^{{c \times k^{2} \times h \times w}}$ is then adjusted using the spatial-channel weight $\Theta$. Many researchers have demonstrated that attention mechanisms [53–55] can effectively adjust local information by identifying key features in an input image and assigning them higher weights. This enables the model to retain finer details. Therefore, the SEKG [52] module is added after the upsampling process, which aims to learn and utilize specific features that are optimal for denoising, thereby enhancing the image quality. Shortcut connections are used to compensate for information distortion caused by upsampling. Additionally, a Parametric Rectified Linear Unit [56] (PReLU) is introduced after convolution to expedite the convergence of the network.

#### DFTM

In recent years, researchers have been diligently working on designing innovative feature fusion modules aimed at seamlessly integrating features from different sources to explore the potential value of data more comprehensively. Zhang et al. [57] proposed a fast and flexible denoising network that seamlessly integrates noise image estimation and noise level estimation to eliminate intricate noise patterns effectively. Gao et al. [58] introduced a multistream denoising network that incorporates a multiscale fusion module to effectively capture noise across various scales. Zhang et al. [50] proposed a transformer-integrated multiencoder network that uses a feature fusion module to compress and fuse features from image, prior, and transformer encoders to eliminate finite-angle artifacts. Based on the insights of these researchers, a DFTM module was designed. As depicted in Fig. 2, the DFTM module is used to fuse and compress diverse features. First, all the features are connected to the middle layer $\left[ {F_{a} ,F_{b} , \cdot \cdot \cdot ,F_{n} } \right] \in R^{{\frac{H}{4}{ \times }\frac{W}{4}{ \times (}256 + 256 + , \cdot \cdot \cdot , + 256)}}$; Subsequently, the intermediate features are condensed into shared features $F_{share} \in R^{{\frac{H}{4}{ \times }\frac{W}{4}{ \times }256}}$ through convolutional layers, and FTM modules with varying depths extract identical features from different sources to obtain shared features at different image levels. This facilitates the mutual complementation of features between different datasets.

Previous studies have indicated that incorporating residual structures [59–61] into models can improve the model performance while minimizing information loss. In this study, to transform the features extracted from a multisource dataset using encoders into shared features, the residual structures are modified and implemented in the experiments. For the FTM (Fig. 3b), four convolution filters are used, and 1 ${ \times }$ 1 convolution is used to fuse the features after global averaging pooling. A sigmoid activation function is used to reduce the feature weight to a range of 0-1, thereby adjusting the importance of the input features. All the channels have a size of 256. To avoid information loss, input features from the original LDCT image are connected to the output. The processing of the DFTM module is illustrated using the following formulas:

5 $$F_{share} { = }C{\text{(cat[}}F_{a} ,F_{b} , \cdot \cdot \cdot ,F_{n} ])$$

6 $$\it F_{SE} = C_{1} PC_{1} (AP(F_{CP} (F_{{{\text{share}}}} )))$$

7 $$\it \it \it F_{DM} = F_{CP} (F_{{{\text{share}}}} ){ \otimes \sigma \{ }F_{SE} [F_{CP} (F_{{{\text{share}}}} )]\} + F_{{{\text{share}}}}$$

where $F_{share}$ represents shared features and $C$ and cat represent 3 ${ \times }$ 3 convolution and concatenation operations, respectively.$F_{CP}$ consists of two 3 ${ \times }$ 3 convolutions, followed by PReLU to extract global residual information.$C_{1}$ and $PC_{1}$ denote a 1 ${ \times }$ 1 convolution layer, PReLU, and a convolution layer of 1 ${ \times }$ 1, respectively.$AP$ represents the average pooling.$\upsigma$ denotes the sigmoid activation function and ${ \otimes }$ represents the element-wise multiplication operation.$F_{DM}$ refers to the final output.

### Loss function

Previous research has indicated that selecting an appropriate loss function can enhance the capability of the model to accurately capture the feature distribution of the data. Moreover, the loss function serves as a metric for assessing the training progress of the model and provides feedback for fine-tuning the predictive parameters and hyperparameters to minimize the loss function and achieve more precise predictions. The least absolute deviation (L1) loss function calculates the absolute difference between the estimated and true values and is insensitive to outliers, which is beneficial for maintaining the model stability when there are exceptional values. The structural similarity index measurement (SSIM) [62] measures the similarity of images from three perspectives: luminance, contrast, and structure. The higher the SSIM value, the more similar the two images. In image-denoising tasks, the SSIM loss function can better preserve the details and texture information of an image, and improve the quality of the reconstructed image. Therefore, in this study, L1 and SSIM have been selected to construct a composite loss function. The formulation of the loss function is expressed in Eq. (8).

8 $$L_{loss} = \alpha L_{1} \left( {\left( {\begin{array}{*{20}c} {I_{ref}^{{\text{a}}} } \\ \begin{gathered} I_{ref}^{b} \\ \cdot \cdot \cdot \\ \end{gathered} \\ {I_{ref}^{n} } \\ \end{array} } \right),\left( {\begin{array}{*{20}c} {I_{pred}^{a} } \\ \begin{gathered} I_{pred}^{b} \\ \cdot \cdot \cdot \\ \end{gathered} \\ {I_{pred}^{n} } \\ \end{array} } \right)} \right) +\uplambda L_{SSIM} \left( {\left( {\begin{array}{*{20}c} {I_{ref}^{a} } \\ \begin{gathered} I_{ref}^{b} \\ \cdot \cdot \cdot \\ \end{gathered} \\ {I_{ref}^{n} } \\ \end{array} } \right),\left( {\begin{array}{*{20}c} {I_{pred}^{a} } \\ \begin{gathered} I_{pred}^{b} \\ \cdot \cdot \cdot \\ \end{gathered} \\ {I_{pred}^{n} } \\ \end{array} } \right)} \right)$$

where $$I_{pred}^{a}$$$$I_{pred}^{b}$$,…,$$I_{pred}^{n}$$ indicate the predicted denoising results from multiple sources, and $$I_{ref}^{a}$$,$$I_{ref}^{b}$$,…,$$I_{ref}^{n}$$ represent NDCT reference images.$$\alpha$$ and $$\uplambda$$ are the hyperparameters of the different loss terms, and $$L_{SSIM}$$ is the SSIM loss function.

In this study, the loss function is primarily influenced by the training process of vertical FL [63]. In the vertical FL training process, the objective function of vertical FL is represented by Eq. (9).

9 $$\mathop {\min }\limits_{{w^{1} ,w^{2} , \cdot \cdot \cdot ,w^{k} }} L = \mathop {\min }\limits_{{w^{1} ,w^{2} , \cdot \cdot \cdot ,w^{k} }} \sum\limits_{k = 1}^{K} {L_{k} \{ f_{{w^{k} }} (x_{i}^{k} ) - y_{i}^{k} \} }$$

where $L = \sum\limits_{k = 1}^{K} {L_{k} }$, $K$ is the total institution,$x_{i}^{k}$, and $y_{i}^{k}$ represent the LDCT image and NDCT image of institution $k$. Each institution has a model $\{ f_{{w^{k} }} \}_{k = 1}^{k}$, and $w^{k}$ is the corresponding weight. The vertical FL is solved for the local parameter $w^{1} ,w^{2} , \cdot \cdot \cdot ,w^{k}$ according to the global loss function $L$ until the network converges. MDFTN also uses a joint loss function to solve the parameter weights. Therefore, the total loss function $L_{total}$ used in this study is given by Eq. (10).

10 $$L_{total} = \mathop {\min }\limits_{{w^{a} ,w^{b} , \cdot \cdot \cdot ,w^{n} }} \sum\limits_{n = a}^{n} {L_{loss} }$$

where $n$ is total institution and $w^{n}$ is the corresponding weight of each source data.

## Results

### Dataset

#### Multisource synthesized clinical dataset

In the experiments, three datasets were utilized to validate the proposed method: the AAPM-Mayo [31], private synthetic clinical [40] and RPLHR-CT [64] datasets. These datasets were acquired using a Siemens CT scanner, ScintCare CT128 scanner (Minfound Medical Co. Ltd., China) and Philips CT devices. The size of each image is 512 ${ \times }$ 512. To confirm the effectiveness of the proposed network, 6000 (2000 ${ \times }$ 3) pairs of normal-dose and corresponding low-dose images were randomly selected from the three LDCT datasets. The training, verification, and testing sets consisted of 4800 (1600 ${ \times }$ 3), 600 (200 ${ \times }$ 3), and 600 (200 ${ \times }$ 3) pairs, respectively. Second, to further test the robustness of the model, a new private dataset with high and low noise levels was added to test the model, which was synthesized using NDCT images from the AAPM-Mayo dataset [31] using the method described in ref. [65] and was only used for the test process. To test the robustness of the model, 200 pairs of images with high and low noise levels were selected randomly. In addition, to further assess the domain-shift problem of the model, an external, independent synthetized dataset was employed in this study, which was used only for the test process and not for the training process. The dataset is a pair of CT-synthesized images obtained by the method in ref. [65] from Siemens CT images with a slice thickness of 5 mm and a tube current of more than 200 mA. For the domain-shift test, 200 pairs were randomly selected.

#### Multisource real clinical dataset

In addition to verifying the effectiveness of the network on the synthesized datasets, validation was also conducted using real clinical LDCT images – Siemens and Minfound clinical datasets–acquired from the Siemens CT scanner and the ScintCare CT128 scanner, which was only used for the test process and was not involved in the training process. The Siemens clinical dataset comprised 10 patients with a total of 2028 LDCT images. The tube current and slice thickness were 65 mA and 1.5 mm, respectively. For the Minfound clinical dataset, 584 LDCT images were obtained from two patients with a tube voltage of 120 KVp, tube current of 80 mA and 40 mA, and slice thicknesses of 1.25 mm and 2.5 mm. The details of the real clinical datasets used in the experiment are listed in Table 1.

**Table 1:** Information of Siemens and Minfound real clinical datasets

Dataset	Patient number	Tube voltage (KVp)	Tube current (mA)	Slice (mm)	Number
Siemens	Patients 1:10	110	65	1.5	2028
Minfound	Patient1	120	80	1.25	192
	Patient2	120	40	1.25	208

### Experimental details

During the training process, 80 ${ \times }$ 80 patches were randomly cropped from the 512 ${ \times }$ 512 LDCT image. The minimum batch size was set to 16. The Adam [66] algorithm was utilized to optimize the network during training. The learning rate was set to 0.0001 with a drop rate of 0.5 for every 20 epochs. The hyperparameters $\alpha$ and λ of the loss function were set to 1 and 0.001, respectively. The network was trained for 100 iterations. The entire experiment was conducted using Python with the PyTorch framework on an NVIDIA TITAN V GPU.

The network was compared with five neural network algorithms: RED-CNN [25], WGAN-VGG [26], WGAN-RAM [67], MAPNN [27], and MINFMCNN [28]. To provide a clearer understanding of these comparison algorithms as well as well as the proposed network, Table 2 presents the operational details, parameters and runtime of each test example for each method. The runtime of a single image is the ratio of the total time to the number of images. The total time is the duration required to compute the testing datasets (600 (200 ${ \times }$ 3)) using the parameters of the trained model.

**Table 2:** Operational details, parameters, and runtimes of the comparison networks

Network model	RED-CNN [25]	WGAN-VGG [26]	WGAN-RAM [67]	MAPNN [27]	MINFMCNN [28]	MDFTN
Mini-batch size	20	20	16	20	10	16
Path size	55 ${ \times }$ 55	80 ${ \times }$ 80	80 ${ \times }$ 80	64 ${ \times }$ 64	80 ${ \times }$ 80	80 ${ \times }$ 80
Training epoch	100	100	100	100	100	100
Learning rate	0.0001	0.0001	0.0001	0.0001	0.0001	0.0001
Optimizer	Adam	Adam	Adam	Adam	Adam	Adam
Parameters	1.84 ${ \times }$ 10^6^	34.07 ${ \times }$ 10^6^	5.56 ${ \times }$ 10^6^	7.72 ${ \times }$ 10^6^	4.41 ${ \times }$ 10^6^	7.18 ${ \times }$ 10^6^
Run times	0.189 s	0.094 s	0.355 s	0.104 s	0.302 s	0.084 s

Given that the proposed network encodes multisource data in a parallel manner, it requires more parameters than the other methods. By contrast, MDFTN has a shorter inference time because multisource data are processed simultaneously. To ensure a fair comparison, all the networks utilized the same training and test datasets as those used in the model. To quantitatively analyze the image quality after denoising, three quality quantitative evaluation indices were employed: peak signal-to-noise ratio (PSNR), structural similarity index measurement (SSIM), and root mean square error (RMSE). PSNR measures the denoising effect and SSIM measures the structural similarity between two images. The larger the PSNR and SSIM values, the closer the results are to the ground truth images, indicating that higher-quality images are produced.

### Experimental results

#### Results of multisource synthesized datasets

**Fig. 4:**
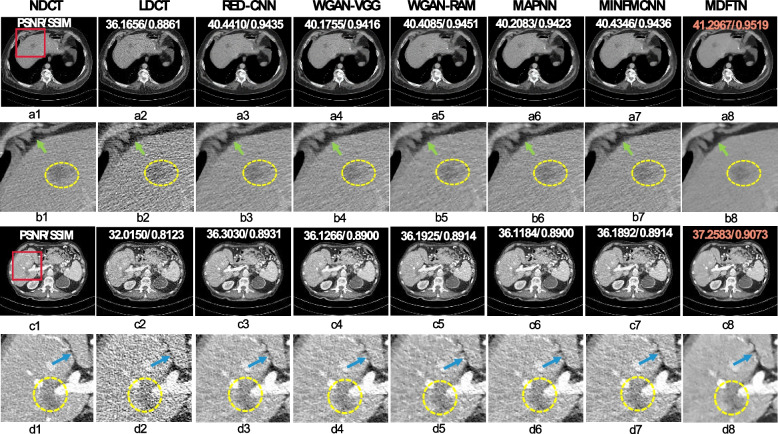
Results and magnified views of data from the AAPM-Mayo synthesized dataset provided for comparison. **a1-d1** NDCT; **a2-d2** LDCT; **a3-d3** RED-CNN; **a4-d4** WGAN-VGG; **a5-d5** WGAN-RAM; **a6-d6** MNPNN; **a7-d7** MINFMCFF; and **a8-d8** MDFTN. The respective ROI for each predicted image is displayed below the image itself. A yellow circle denotes a metastasis, whereas blue and green arrows indicate fine structures. The orange number signifies the highest quantitative index. The display window is set at [-160, 240] HU

**Fig. 5:**
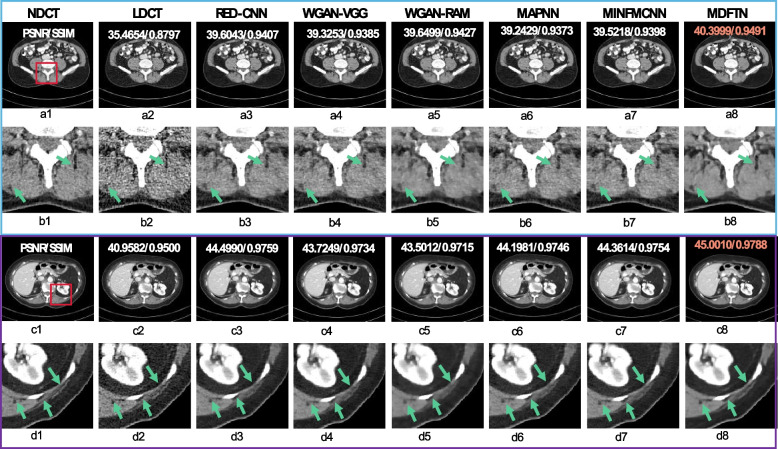
Results and magnified views of data from a private synthesized dataset with different noise levels are provided for comparison. **a1-b8** High noise levels; **c1-d8** Low noise levels; **a1-d1** NDCT; **a2-d2** LDCT; **a3-d3** RED-CNN; **a4-d4** WGAN-VGG; **a5-d5** WGAN-RAM; **a6-d6** MNPNN; **a7-d7** MINFMCFF; and **a8-d8** MDFTN. The corresponding ROI for each predicted image is shown below itself. The green arrows indicate some fine structure regions. The orange number represents the highest quantitative index. The display window is [-160, 240] HU

**Fig. 6:**
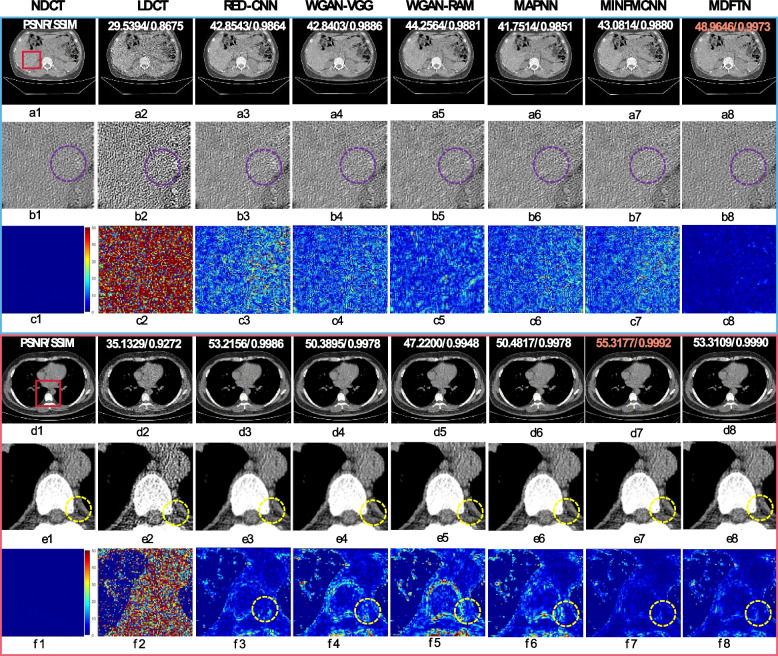
Results and magnified views of data from the synthesized Minfound dataset and RPLHR-CT dataset are provided for comparison. The corresponding ROI for each predicted image is shown below itself. The difference images between the predicted results of different methods and NDCT are labeled as (**c1-c8**) and (**f1-f8**), respectively. The orange number represents the highest quantitative index. The purple circles denote flat regions for noise suppression analysis, while the yellow circles highlight detailed structures for visual comparison. The display window range is set at [-160, 240] HU

This subsection presents the visual and quantitative results of the different methods on multiple source datasets. Figures 4, 5 and 6 show the visual results for each network and the corresponding quantitative results (PSNR/SSIM). Orange numbers indicate the highest quantitative indices. Figure 4 displays the visual results and enlarged views of the proposed network (MDFTN) and the comparison algorithms on the AAPM-Mayo synthesized dataset. Two representative lesion images were selected, and the corresponding zoomed-in regions of interest (ROIs) (indicated by red rectangles) were extracted from the predicted results and true images. The yellow circles denote metastases, whereas the blue and green arrows denote fine structures. From Fig. 4b1-b8, it is evident that all the methods are capable of reducing the noise level of the images and enhancing the distinction of metastasis to some extent. However, in the edge region indicated by the green arrow, the algorithm demonstrates clear advantages in preserving the details. In cases where the CT images contain more tissue structures, such as abdominal CT images, Fig. 4c2 exhibits more complex noise and artifacts than Fig. 4a2 in the LDCT image. It can be seen from Fig. 4c1-c8 that the image processed by the MDFTN exhibits clearer contrast than the other algorithms, particularly in the ROIs of Fig. 4d3-d8. When compared to the NDCT images, the resulting image processed by the WGAN-RAM (Fig. 4d5) has more noise and artifacts, resulting in poor resolution near the ligamentum teres in the liver (indicated by the blue arrow). At this noise level, the overall images of the ROIs obtained by the WGAN-VGG (Fig. 4d4), MAPNN (Fig. 4d6), and MINFMCNN (Fig. 4d7) are relatively similar, but the details appear blurry. Notably, the detailed features of the ligamentum teres indicated by the blue arrows demonstrate that the MDFTN (Fig. 4d8) produces clearer results than the RED-CNN (Fig. 4d3). Overall, the MDFTN method achieves a more detailed structure and quantitative index, as shown in Fig. 4a8 and Fig. 4c8.

To further verify the robustness of the trained model in processing LDCT images with different noise levels, the test results of the model using a privately synthesized dataset with high and low noise levels are also presented. The dataset was obtained by using the method described in ref. [65]. Figure 5 shows the visualized results and enlarged views of MDFTN and the comparison algorithms. It is evident that Figs. 5a1-b8 exhibit more noise than Figs. 5c1-d8. Upon examining Fig. 5, it is apparent that the proposed MDFTN effectively suppresses noise while preserving the detailed structures (indicated by green arrows) under both high and low noise levels. Tables 3 and 4 present the quantitative outcomes of the different algorithms on privately synthesized datasets with high and low noise levels, respectively. It was evident that MDFTN delivers the highest PSNR and SSIM scores.

**Table 3:** Quantitative results (mean ± SD) of different methods on private synthesized dataset with high noise levels

	LDCT	RED-CNN	WGAN-VGG	WGAN-RAM	MAPNN	MINFMCNN	MDFTN
Avg_PSNR	33.6662 ± 1.1778	37.8209 ± 1.0990	37.5914 ± 1.0896	37.9155 ± 1.0734	37.5363 ± 1.0767	37.6866 ± 1.1346	**38.7281 ± 1.1256**
Avg_SSIM	0.8375 ± 0.0270	0.9134 ± 0.0170	0.9103 ± 0.0176	0.9160 ± 0.0167	0.9096 ± 0.0175	0.9114 ± 0.0178	**0.9263 ± 0.0121**
Avg_RMSE	0.0092 ± 0.0013	0.0057 ± 0.0007	0.0058 ± 0.0007	0.0056 ± 0.0007	0.0059 ± 0.0007	0.0058 ± 0.0006	**0.0051 ± 0.0007**

Note: Black bold numbers represent optimal results

**Table 4:** Quantitative results (mean ± SD) of different methods on private synthesized dataset with low noise levels

	LDCT	RED-CNN	WGAN-VGG	WGAN-RAM	MAPNN	MINFMCNN	MDFTN
Avg_PSNR	41.9816 ± 1.8122	44.8241 ± 1.2118	44.0980 ± 1.1056	43.5910 ± 0.7866	44.6007 ± 1.2638	44.8517 ± 1.2941	**45.2988 ± 1.3010**
Avg_SSIM	0.9588 ± 0.0117	0.9787 ± 0.0048	0.9763 ± 0.0051	0.9744 ± 0.0049	0.9779 ± 0.0052	0.9786 ± 0.0051	**0.9811 ± 0.0052**
Avg_RMSE	0.0036 ± 0.0006	0.0025 ± 0.0003	0.0028 ± 0.0003	0.0029 ± 0.0003	0.0026 ± 0.0004	0.0025 ± 0.0003	**0.0024 ± 0.0003**

Note: Black bold numbers represent optimal results

Figure 6 shows the visual results of the proposed network (MDFTN) along with the comparison algorithms for the synthesized Minfound and RPLHR-CT datasets. It includes enlarged views and difference maps for a detailed analysis. From Fig. 6, it is apparent that the visual impact of the human eye on image visualization is not significant. Therefore, difference images were generated between the predicted results processed by the different methods and the NDCT, as shown in Figs. 6c1-c8 and f1-f8. Figures 6a1-c8 present the visualization results for the synthesized Minfound dataset. Upon observing Figs. 6b1-b8 and c1-c8, all the methods effectively removed noise from the low-frequency region (indicated by the purple circle). However, the proposed method demonstrates a more significant denoising effect, resulting in a higher quantitative index. Figures 6d1-f8 show the visualization results of the RPLHR-CT dataset, with a specific focus on the skeletal region. In terms of image detail, the WGAN-RAM (Figs. 6e5 and f5) appears to be more blurred than the other algorithms. Upon analyzing the difference image in the bottom row of Fig. 6, it is evident that RED-CNN (Fig. 6f3), MINFMCNN (Fig. 6f7), and MDFTN (Fig. 6f8) demonstrate similar structure preservation. However, MINFMCNN and MDFTN exhibit fewer residual details, suggesting that they possess superior detail-preservation capabilities, as highlighted by the yellow circles.

In addition to visual comparisons with other algorithms, the PSNR/SSIM/RMSE metrics were utilized to quantitatively evaluate the proposed MDFTN and other methods. Tables 5, 6 and 7 show the PSNR, SSIM, and RMSE measures for the comparison methods applied to the different datasets. Compared with the LDCT index, all methods improved the PSNR and SSIM indices to some extent and reduced the RMSE index. It is evident that MDFTN achieves the highest PSNR and SSIM results and the lowest RMSE for both the AAPM-Mayo and the synthesized Minfound datasets. On the RPLHR-CT dataset, the MINFMCNN achieves the highest PSNR and SSIM results, followed by the proposed algorithm. However, for the other two datasets, MINFMCNN performs worse than the proposed method. Therefore, it is evident that the proposed method exhibits better generalization and robustness than the other algorithms. This is because MDFTN utilizes collaborative training across various datasets, leveraging the complementary advantages of multisource data distribution to enhance its adaptability and improve its generalization. In summary, the proposed MDFTN demonstrates promising performance in terms of noise reduction and structural preservation when processing multiple source datasets simultaneously.

**Table 5:** PSNR (mean ± SD) metrics of different methods on different source datasets

Avg_PSNR	LDCT	RED-CNN	WGAN-VGG	WGAN-RAM	MAPNN	MINFMCNN	MDFTN
AAPM-Mayo	36.1466 ± 1.0673	40.0503 ± 0.8310	39.6790 ± 0.7880	39.8985 ± 0.8001	39.77569 ± 0.8124	39.9636 ± 0.8670	**40.7208** ± 0.7669
Synthesized Minfound	31.1490 ± 1.1769	43.2322 ± 0.9701	42.6747 ± 0.9986	43.9589 ± 0.9680	41.8829 ± 0.9999	43.2792 ± 0.9448	**47.7294** ± 2.0596
RPLHR-CT	34.2046 ± 1.9651	52.8923 ± 0.6776	50.3037 ± 0.6559	47.4452 ± 0.5689	50.2550 ± 0.7264	**55.7721** ± 0.8501	53.8253 ± 0.8344

Note: Black bold numbers represent the optimal results. Assuming an error in the calculation of the PSNR, standard is equal to the calculated standard deviation of the PSNR of a single image and the mean PSNR. The SSIM/RMSE standard was the same as described above

**Table 6:** SSIM (mean ± SD) metrics of different methods on different source datasets

Avg_SSIM	LDCT	RED-CNN	WGAN-VGG	WGAN-RAM	MAPNN	MINFMCNN	MDFTN
AAPM-Mayo	0.8958 ± 0.0195	0.9469 ± 0.0097	0.9438 ± 0.0080	0.9471 ± 0.0094	0.9443 ± 0.0102	0.9459 ± 0.0102	**0.9532** ± 0.0081
Synthesized Minfound	0.8890 ± 0.0175	0.9883 ± 0.0021	0.9438 ± 0.0101	0.9913 ± 0.0018	0.9886 ± 0.0019	0.9893 ± 0.0018	**0.9964** ± 0.0012
RPLHR-CT	0.9150 ± 0.0286	0.9986 ± 0.0004	0.9896 ± 0.0014	0.9949 ± 0.0080	0.9977 ± 0.0005	**0.9993** ± 0.0001	0.9991 ± 0.0002

Note: Black bold numbers represent optimal results

**Table 7:** RMSE (mean ± SD) metrics of different methods on different source datasets

Avg_RMSE	LDCT	RED-CNN	WGAN-VGG	WGAN-RAM	MAPNN	MINFMCNN	MDFTN
AAPM-Mayo	0.0069 ± 0.0008	0.0044 ± 0.0004	0.0046 ± 0.0004	0.0045 ± 0.0004	0.0045 ± 0.0004	0.0044 ± 0.0004	**0.0041** ± 0.0004
Synthesized Minfound	0.0123 ± 0.0017	0.0030 ± 0.0003	0.0033 ± 0.0004	0.0028 ± 0.0003	0.0036 ± 0.0004	0.0030 ± 0.0003	**0.0019** ± 0.0005
RPLHR-CT	0.0088 ± 0.0021	0.0010 ± 0.0001	0.0013 ± 0.0001	0.0019 ± 0.0001	0.0014 ± 0.0001	**0.0007** ± 0.0001	0.0009 ± 0.0001

Note: Black bold numbers represent optimal results

#### Results of independent synthesized datasets

**Fig. 7:**
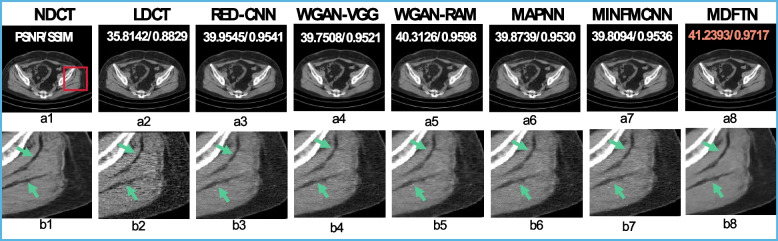
Results and magnified views of data from independent synthesized datasets are provided for comparison. **a1-b1** NDCT; **a2-b2** LDCT; **a3-b3** RED-CNN; **a4-b4** WGAN-VGG; **a5-b5** WGAN-RAM; **a6-b6** MNPNN; **a7-b7** MINFMCFF; and **a8-b8** MDFTN. The corresponding ROI for each predicted image is shown below itself. The green arrows indicate boundary texture. The orange number represents the highest quantitative index. The display window is [-160, 240] HU

To further assess the domain-shift problem of the model, an external independently synthesized dataset was employed in this study. Figure 7 displays the visual results and enlarged views of the proposed network (MDFTN) and comparison algorithms on independently synthesized datasets. It can be seen from Figs. 7b1-b8 that the image processed by MDFTN exhibits remarkable similarity to the real image compared to the other algorithms, particularly in the border region indicated by the green arrow. Table 8 presents the quantitative results of the different models for the independently synthesized datasets. It is evident that MDFTN achieves the highest PSNR and SSIM results and the lowest RMSE for the independently synthesized datasets.

**Table 8:** Quantitative results (mean ± SD) of different methods on independent synthesized datasets

	LDCT	RED-CNN	WGAN-VGG	WGAN-RAM	MAPNN	MINFMCNN	MDFTN
Avg_PSNR	38.5157 ± 2.1155	41.0655 ± 0.7906	40.8018 ± 0.7404	41.0503 ± 0.5547	40.9922 ± 0.8125	40.9026 ± 0.7727	**41.8939 ± 0.8019**
Avg_SSIM	0.9314 ± 0.0383	0.9704 ± 0.0118	0.9685 ± 0.0121	0.9707 ± 0.0075	0.9698 ± 0.0123	0.9703 ± 0.0121	**0.9799 ± 0.0115**
Avg_RMSE	0.0054 ± 0.0013	0.0039 ± 0.0004	0.0040 ± 0.0003	0.0039 ± 0.0003	0.0039 ± 0.0004	0.0040 ± 0.0004	**0.0035 ± 0.0004**

Note: Black bold numbers represent optimal results

#### Results of multisource real clinical datasets

**Fig. 8:**
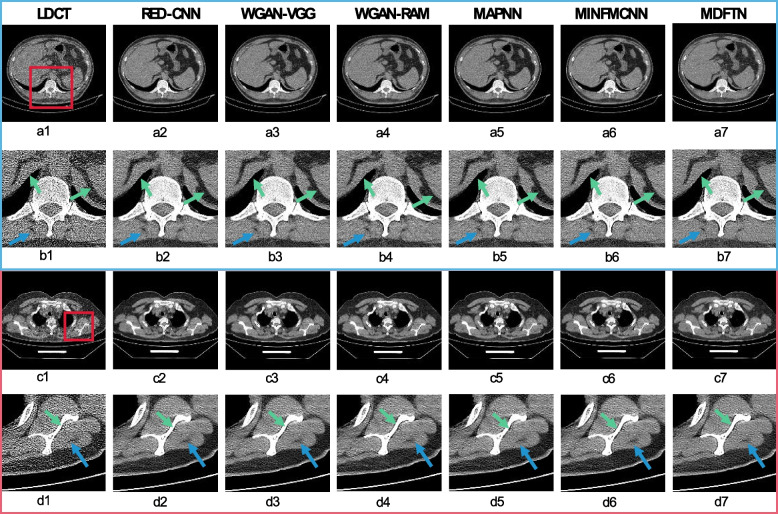
Results and magnified views of data from the real Siemens LDCT dataset and the Minfound LDCT dataset are provided for comparison. **a1-d1** LDCT; **a2-d2** RED-CNN; **a3-d3** WGAN-VGG; **a4-d4** WGAN-RAM; **a5-d5** MNPNN; **a6-d6** MINFMCFF; and **a7-d7** MDFTN. The corresponding ROI for each predicted image is shown below itself. The blue arrows denote flat regions while green arrows indicate high-contrast edge structures. The display window is [-160, 240] HU

Considering the presence of feature distribution differences between the simulated and real clinical LDCT images, the universality and stability of the network were further verified using real Siemens and Minfound LDCT data. To ensure flexibility, clinical data were exclusively used for network testing by employing the optimal parameters trained on the synthesized datasets. Figures 8a1-b7 show the visualization results of the proposed network (MDFTN) and the comparison algorithms on real Siemens clinical LDCT data, and Figs. 8c1-d7 illustrate the visualization results of the Minfound LDCT data, with corresponding zoomed ROIs (represented by red rectangles) cropped from the predicted images. It can be observed from Fig. 8a1-d1 that the LDCT images contain significant amounts of noise and artifacts. Although all algorithms can improve image quality, they still have limitations. In comparison with the overall image in Figs. 8a1-a7, the result predicted by the MDFTN algorithm is more favorable for doctors owing to fewer residual artifacts. As depicted in Figs. 8b2-b7, the WGAN-VGG (Fig. 8b3), WGAN-RAM (Fig. 8b4), and MAPNN (Fig. 8b5) still exhibit some noise and artifacts, potentially impacting doctors’ observations during clinical diagnosis. MDFTN effectively removes a significant amount of noise and artifacts, as indicated by the blue arrows. The marginal areas of the liver and spleen, indicated by green arrows (Fig. 8b7) are clearer than those of the other algorithms. On the real Minfound LDCT data (Figs. 8d2-d7), MDFTN (Fig. 8d7) exhibits a sharper contrast than MAPNN (Fig. 8d5) in the bone boundary region represented by the green arrow. In the low-frequency region indicated by the blue arrow, RED-CNN (Fig. 8d2), WGAN-VGG (Fig. 8d3), and WGAN-RAM (Fig. 8d4) still contain some artifacts, resulting in poor contrast observation in the low-frequency region. The results for MAPNN (Fig. 8d5), MINFMCNN (Fig. 8d6), and MDFTN (Fig. 8d7) are similar in the area indicated by the blue arrow. In summary, the results of clinical trials on a real Siemens LDCT dataset and the Minfound LDCT dataset demonstrate that MDFTN has certain advantages in noise removal and artifact suppression compared with the other algorithms.

### Ablation experiment

In this subsection, the ablation studies of the proposed network are described. First, the denoising performance of the proposed network are examined in the single-source model, and then the effectiveness of the FTM and attention mechanism SEKG [52] are verified. Third, the versatility of the proposed network was assessed using the RED-CNN network.

#### Results of single-source MDFTN

**Fig. 9:**
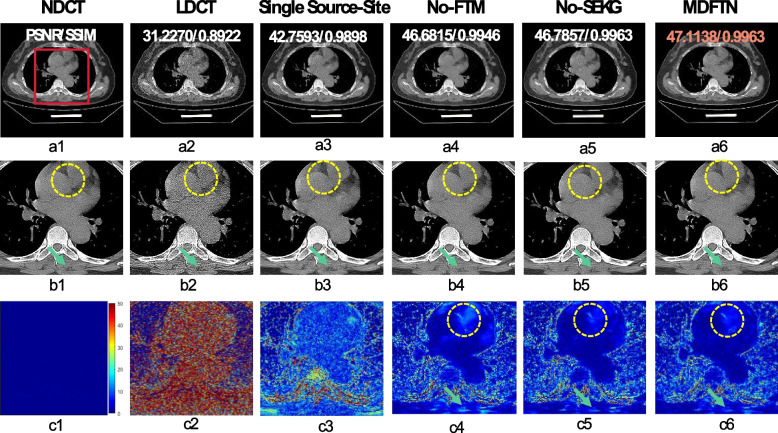
Ablation results and magnified views of data from the synthesized Minfound dataset are used for the single-source-site, without the FTM and without the attention mechanism SEKG. **a1-c1** NDCT; **a2-c2** LDCT; **a3-c3** Sing Source-Site; **a4-c4** No-FTM; **a5-c5** No-SEKG; **a6-c6** MDFTN. No-FTM means without the FTM. No-SEKG means without the attention mechanism SEKG [52]. The corresponding ROI to each predicted image is shown below itself. **c1-c6** denote the difference images between the predicted results of different methods and NDCT. The orange number represents the highest quantitative index. The yellow circles and green arrows indicate subtle details within low-contrast structured regions. The display window range is set at [-160, 240] HU

The full MDFTN framework was used to implement collaborative training to improve the denoising performance of the LDCT. In this study, a single-source MDFTN was employed to assess the effects of collaborative training. Figure 9 shows the visualization results of the MDFTN with and without different components on the synthesized Minfound dataset. Figures 9a3-c3 and 9a6-c6 show the denoising results of the single- and multisource MDFTN, respectively. In the first row, compared to Figs. 9a3 and a6, the full MDFTN (Fig. 9a6) appears to exhibit less noise and a higher evaluation index than the single-source MDFTN (Fig. 9a3). Upon examining the flat regions highlighted by yellow circles and green arrows, the complete MDFTN model demonstrates superior preservation of details in the soft tissue areas. Table 9 presents the averages of the qualitative results. These quantitative results demonstrate that the full network exhibits significant improvements in terms of PSNR, SSIM, and RMSE. The above results indicate that the full network can not only simultaneously process LDCT images from multiple sources but also effectively integrate advantageous features from different datasets during the training process.

**Table 9:** Ablation results of quantitative index (mean ± SD) for single-source-site, no-FTM and attention mechanism on three synthesized datasets

Dataset		Avg_PSNR	Avg_SSIM	Avg_RMSE
LDCT	AAPM-Mayo	36.1466 ± 1.0673	0.8958 ± 0.0195	0.0069 ± 0.0008
	Minfound	31.1498 ± 1.1769	0.8890 ± 0.0175	0.0123 ± 0.0017
	RPLHR-CT	34.2046 ± 1.9651	0.9150 ± 0.0286	0.0088 ± 0.0021
Single source-site	AAPM-Mayo	39.9046 ± 0.8573	0.9454 ± 0.0102	0.0045 ± 0.0004
	Minfound	43.2078 ± 0.9898	0.9895 ± 0.0017	0.0031 ± 0.0003
	RPLHR-CT	53.5080 ± 0.8858	0.9989 ± 0.0002	0.0009 ± 0.0001
No-FTM	AAPM-Mayo	40.6361 ± 0.7474	0.9525 ± 0.0081	**0.0041** ± 0.0003
	Minfound	47.3316 ± 2.0064	0.9950 ± 0.0017	**0.0019** ± 0.0005
	RPLHR-CT	53.2662 ± 0.8078	0.9989 ± 0.0002	0.0010 ± 0.0001
No-SEKG	AAPM-Mayo	40.6880 ± 0.7388	0.9529 ± 0.0080	**0.0041** ± 0.0003
	Minfound	47.4729 ± 2.0401	**0.9967** ± 0.0011	**0.0019** ± 0.0005
	RPLHR-CT	53.4101 ± 0.7939	0.9989 ± 0.0002	**0.0009** ± 0.0001
MDFTN	AAPM-Mayo	**40.7208** ± 0.7669	**0.9532** ± 0.0081	**0.0041** ± 0.0004
	Minfound	**47.7294** ± 2.0596	0.9964 ± 0.0012	**0.0019** ± 0.0005
	RPLHR-CT	**53.8253** ± 0.8344	**0.9991** ± 0.0002	**0.0009** ± 0.0001

Note: Black bold numbers represent the optimal results. A single source site refers to a single coding structure. No-FTM means without the feature transformation module. No-SEKG indicates no attention mechanism (SEKG)

#### Effectiveness of FTM and SEKG

First, the effectiveness of the FTM are verified in the model. Figures 9a4-c4 show the visual results, enlarged views, and difference images of the network without the FTM on the synthesized Minfound dataset. As can be observed from the MDFTN (Fig. 9b6) and No-FTM (Fig. 9b4), the full network clearly emphasizes the edges in the low-frequency region, as indicated by the green arrow, whereas the details in No-FTM (Fig. 9b4) appear unclear. In the third row of the difference images, it can be seen that the results of the full network are closer to the actual conditions in the heart region, marked by the yellow circle. The quantitative results for no-FTM and MDFTN in Table 9 also demonstrate the beneficial impact of FTM on the overall network.

Subsequently, the effectiveness of the SEKG [52] introduced in the decoder was assessed on three synthesized datasets. Figures 9a5-c5 illustrate the visual results, enlarged views, and difference images of the network without SEKG on the synthesized Minfound dataset. The denoising result of MDFTN (Fig. 9c6) is closer to the background image than that of No-SEKG (Fig. 9c5) in the area indicated by the green arrow. Table 9 shows that the inclusion of the attention mechanism SEKG can improve the quantitative results of the PSNR and SSIM for the network across the three datasets. These results demonstrate that SEKG enhances the performance of the overall network.

#### The versatility of the proposed network framework

**Fig. 10:**
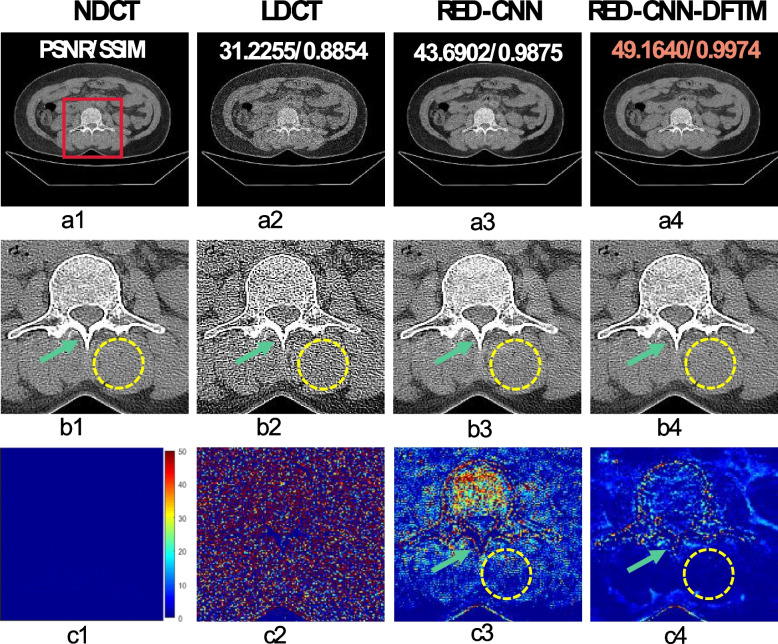
Ablation results and magnified views of data from the synthesized Minfound dataset are used to verify the versatility of the proposed network framework. **a1-c1** NDCT; **a2-c2** LDCT; **a3-c3** RED-CNN; **a4-c4** RED-CNN-DFTM. The corresponding ROI to each predicted image is shown below itself. **c1-c4** denote difference images between the predicted results of different methods and NDCT. The orange number represents the highest quantitative index. The yellow circles indicate the low-density region, while the green arrows indicate the high-contrast region. The display window is [-160, 240] HU

To further verify the versatility of the proposed network framework, the RED-CNN network was incorporated into the framework, resulting in RED-CNN-DFTM. Figure 10 shows the visualization results of the original RED-CNN and the modified RED-CNN (RED-CNN-DFTM) on the synthesized Minfound dataset. Based on the visual observation in Fig. 10, the RED-CNN-DFTM (Fig. 10b4) prediction results are considerably lower than the noise level of the RED-CNN (Fig. 10b3). Moreover, the visibility of the low-density region marked by the yellow circle demonstrates a noticeable improvement in the denoising performance of RED-CNN. In terms of high-contrast edge details, RED-CNN-DFTM outperforms RED-CNN in distinguishing the bone boundaries, as indicated by the green arrows. Compared with the difference map of RED-CNN (Fig. 10c3), RED-CNN-DFTM (Fig. 10c4) exhibits a closer resemblance to the real image in terms of noise and textured background. To further observe the image-denoising performance across the three datasets, Table 10 presents the average quantitative results of RED-CNN-DFTM and RED-CNN. RED-CNN-DFTM improved the PSNR and SSIM indicators of the images compared to RED-CNN. The above results show that the collaborative learning network architecture is indeed helpful for the simultaneous denoising of multisource datasets while achieving superior denoising performance.

**Table 10:** Ablation results of quantitative index (mean ± SD) are used to verify the versatility of the proposed network framework on three synthesized datasets

Dataset		Avg_PSNR	Avg_SSIM	Avg_RMSE
LDCT	AAPM-Mayo	36.1466 ± 1.0673	0.8958 ± 0.0195	0.0069 ± 0.0008
	Minfound	31.1498 ± 1.1769	0.8890 ± 0.0175	0.0123 ± 0.0017
	RPLHR-CT	34.2046 ± 1.9651	0.9150 ± 0.0286	0.0088 ± 0.0021
RED-CNN	AAPM-Mayo	40.0503 ± 0.8310	0.9469 ± 0.0097	0.0044 ± 0.0004
	Minfound	43.2322 ± 0.9701	0.9883 ± 0.0021	0.0030 ± 0.0003
	RPLHR-CT	52.8923 ± 0.6776	0.9986 ± 0.0004	0.0010 ± 0.0001
RED-CNN-DFTM	AAPM-Mayo	**40.4398** ± 0.7257	**0.9512** ± 0.0082	**0.0042** ± 0.0003
	Minfound	**47.5038** ± 1.5820	**0.9957** ± 0.0015	**0.0019** ± 0.0004
	RPLHR-CT	**52.9749** ± 0.6150	**0.9984** ± 0.0004	**0.0010** ± 0.0001

Note: Black bold numbers represent optimal results

### Convergence analysis

**Fig. 11:**
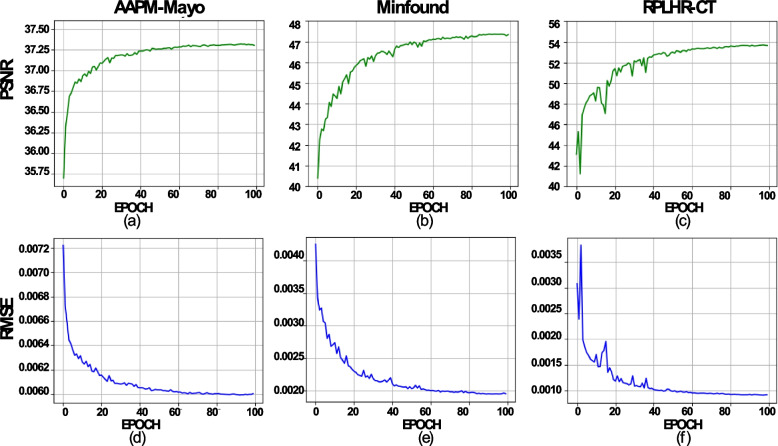
Plots of PSNR and RMSE values *vs* the number of epochs during the training of the MDFTN network models

Because images produced by CT devices from different manufacturers exhibit distinct data distributions, it is crucial to assess the convergence of the MDFTN network, which is jointly trained on multiple source datasets simultaneously. Figure 11 shows a comparison of the convergence rates of the PSNR and RMSE as functions of the epoch number during training on the multisource datasets. As depicted in Figs. 11a, b, c, the PSNR increases rapidly in the initial stages of training, followed by a gradual increase until it stabilizes after the 40th epoch. The RMSE follows a similar trend, stabilizing after the 40th epoch. As shown in Figs. 11c and f, the PSNR and RMSE values exhibit substantial fluctuations during the initial training stage of the RPLHR-CT dataset. This is primarily attributed to the distinct dataset variations when the network updates its parameters. However, it is noteworthy that the network tends to converge after 40 epochs. These findings effectively validate the capability of the MDFTN to simultaneously train on multiple source datasets.

### Hyperparameter analysis

In this subsection, the impact of the loss function hyperparameter λ value in Eq. (8) are investigated on the network’s performance. Table 11 presents the quantitative results obtained by training λ values in [0, 1, 0.1, 0.01, 0.005, 0.001, 0.0001] on the multisource datasets. Through comparison, it is observed that the highest average PSNR and SSIM results are achieved when λ = 0.001. As a result, the loss-function hyperparameter λ = 0.001was chosen.

**Table 11:** Quantitative results (mean ± SD) of weight hyperparameter λ values on the multisource datasets

Evaluation PSNR/SSIM	Dataset		
	AAPM-Mayo	Minfound	RPLHR-CT
λ = 0	40.6961 ± 0.7567/0.9530 ± 0.0082	47.6388 ± 2.0021/0.9967 ± 0.0012	53.5510 ± 0.7708/0.9990 ± 0.0002
λ = 1	40.5950 ± 0.7661/0.9524 ± 0.0082	47.3720 ± 1.8954/0.9960 ± 0.0014	53.0686 ± 0.7170/0.9988 ± 0.0011
λ = 0.1	40.6104 ± 0.7472/0.9524 ± 0.0082	47.2466 ± 1.9608/0.9959 ± 0.0015	53.4677 ± 0.7721/0.9990 ± 0.0002
λ = 0.01	40.6907 ± 0.7570/0.9530 ± 0.0082	47.5967 ± 2.0119/0.9967 ± 0.0011	53.7422 ± 0.7570/0.9990 ± 0.0002
λ = 0.005	40.6851 ± 0.7587/0.9529 ± 0.0081	47.4122 ± 1.9659/0.9954 ± 0.0015	53.7106 ± 0.7158/0.9990 ± 0.0002
λ = 0.001	**40.7208 ± 0.7669/0.9532 ± 0.0081**	**47.7294 ± 2.0596/0.9964 ± 0.0012**	**53.8253 ± 0.8344/0.9991 ± 0.0002**
λ = 0.0001	40.7025 ± 0.7570/0.9530 ± 0.0082	47.5610 ± 2.0016/0.9971 ± 0.0012	53.5388 ± 0.7702/0.9990 ± 0.0002

Note: Black bold numbers represent optimal results

## Discussion

Despite the widespread use of DL models in medical imaging, they have a limited ability to simultaneously process LDCT images from multiple sources by relying solely on a specific dataset. In practical clinical applications, specific networks are used to process CT data from different manufacturers, which may restrict the universality of the model. To address these issues, a learning-once model is presented that efficiently processes multisource LDCT images, allowing the network model to better process data from diverse imaging sources. Based on this, a novel MDFTN is proposed to improve LDCT imaging performance for multisource data. The proposed MDFTN comprises multiple encoders and decoders along with a DFTM. During forward propagation in network training, parallel encoders extract distinct features from their respective data sources, whereas DFTM facilitates the mutual enhancement of multisource data features. In the backward propagation phase of network training, joint loss functions are utilized to calculate the gradient of each layer and subsequently update all the network weights accordingly. Through collaborative training, the proposed MDFTN leverages the complementary advantages of multisource data distribution to enhance its adaptability and generalization.

Given the flexibility of MDFTN, the proposed network framework can be expanded to accommodate various multitask LDCT denoising applications. Unlike previous denoising methods that rely solely on specific datasets, the collaborative training mechanism augments the model’s capacity for generalization across a range of datasets. Furthermore, when addressing LDCT denoising issues across multiple institutions, privacy and security concerns regarding patient data may arise. In such cases, the DFTM can be removed and a shared global model introduced to render MDFTN more suitable for privacy-protection imaging scenarios. Although the proposed MDFTN offers several advantages, it has certain limitations. First, when the DFTM combines and extracts shared features from each encoder, it may lose some globally significant information as the module depth increases. This is because, as the depth of the module increases, gradient disappearance or gradient explosion may occur during backpropagation, making it difficult for the model to learn global information. Secondly, given the varying data distributions across different anatomical sites and CT devices, CT clinical scan data are significantly complex. In this study, the dataset for experimental verification was mainly for the chest and abdominal sections, and more sections need to be applied to the model. In the future more effective deep supervision and fusion methods, will be explored to enhance the performance. In particular, an auxiliary loss function will be incorporated into the intermediate layers of the network, thereby enabling the model to consider both global and local information during training. This approach has the potential to significantly improve the model’s understanding of the data, leading to more accurate and comprehensive results. Secondly, it is crucial to broaden the clinical datasets for LDCT denoising, specifically by incorporating multidose, multiprotocol, and multianatomical site datasets. In addition, considering the significance of LDCT image denoising in clinical practice, the potential applications of the proposed method in real clinical settings are discussed and the challenges that it may face. Although MDFTN can help doctors achieve rapid and precise diagnoses of various diseases by concurrently processing data from different hospitals, it still faces several challenges that need to be addressed. The accurate amassing of paired data is essential for training highly generalized LDCT denoising models. However, obtaining a substantial number of high-quality, precisely matched CT images remains a significant challenge. Therefore, a critical future task is to enhance the capability of the model to denoise real clinical unlabeled LDCT images by designing innovative domain-adversarial loss functions.

## Conclusions

This study aims to address the challenges posed by the different distributions of multisource data and data scarcity through the utilization of DL. To address this problem, a learning-once model is proposed that incorporates multisource encoders and a DFTM module. This model allows the simultaneous processing of multisource data and outperforms a single model with continuous learning. Through collaborative training, the proposed MDFTN network effectively leverages the complementary advantages of the features presented in multisource data, resulting in improved imaging performance and generalization for multisource image denoising. Numerous experiments were conducted on two public datasets and one local dataset, demonstrating that the proposed network model can simultaneously process multisource data while effectively suppressing noise and preserving fine structures.

## Data Availability

Synthesized Clinical Datasets are about the AAPM-Mayo dataset, the private synthetic clinical dataset and the RPLHR-CT dataset. Where AAPM-Mayo dataset is obtained at [31] http://www.aapm.org/GrandChallenge/LowDoseCT/, private synthetic clinical dataset comes from [40] 10.1109/TMI.2023.3261822, and RPLHR-CT dataset comes from [64] 10.1007/978-3-031-16446-0_33. Clinical data are not publicly available because they contain private patient health information. Data supporting the findings of the present study are available upon reasonable request.

## Abbreviations

CT: Computed tomography
DFTM: Deep feature transformation module
DL: Deep learning
FL: Federated learning
GMM: Gaussian mixture model
LDCT: Low-dose computed tomography
MAP-NN: Modularized adaptive processing neural network
MDFTN: Multi-encoder deep feature transformation network
NDCT: Normal-dose computed tomography
PReLU: Parametric Rectified Linear Unit
PSNR: Peak signal-to-noise ratio
SEKG: Spatially enhanced kernel generation
SSIM: Structural similarity index measurement
RMSE: Root mean square error
ROIs: Regions of interest
